# Cisplatin Exposure Alters the Effects of Honokiol on Genes Involved in Outer Hair Cell Physiological Function

**DOI:** 10.64898/2026.10.22.761060

**Published:** 2026-10-24

**Authors:** Chail Koo, Xiaodong Tan

## Abstract

Outer hair cell (OHC) loss occurs in cisplatin-induced hearing loss (CIHL). We previously reported that honokiol protects against CIHL and mitigates cisplatin-induced transcriptomic changes in OHCs. Here, we re-analyzed OHC RNA-seq to determine whether honokiol differentially affects genes involved in OHC physiological function depending on cisplatin exposure. Twenty-two genes involved in mechanotransduction and hair bundle function, membrane potential and ion homeostasis, electromotility, and efferent regulation were selected. Among 20 genes with available interaction statistics, 16 showed significant Honokiol × Cisplatin interactions, of which 15 were negative. Notably, in the presence of cisplatin, honokiol downregulated several genes associated with mechanotransduction (MET) machinery, including *Lhfpl5, Tmc1, Cib2* , and *Tomt* . Because MET channels have been implicated in cisplatin’s entry into hair cells, these changes raise the possibility that altered MET machinery may contribute to honokiol-mediated protection against CIHL. However, whether these changes decrease cisplatin uptake remains to be determined.

